# Sleep-Disordered Breathing and Associated Comorbidities among Preschool-Aged Children with Down Syndrome

**DOI:** 10.3390/children11060651

**Published:** 2024-05-28

**Authors:** Tessa K. Kolstad, Lourdes M. DelRosso, Mary Anne Tablizo, Manisha Witmans, Yeilim Cho, Michelle Sobremonte-King

**Affiliations:** 1 School of Nursing, University of Washington, Seattle, WA 98195, USA; tessa.kolstad@seattlechildrens.org; 2 Department of Internal Medicine, School of Medcine, University of California San Francisco, Fresno, CA 94143, USA; lourdes.delrosso@ucsf.edu (L.M.D.); mtablizo@stanford.edu (M.A.T.); 3 Division of Pulmonology and Sleep Medicine, School of Medicine, Stanford University, Palo Alto, CA 94305, USA; 4 Department of Pediatrics, University of Alberta, Edmonton, AB T6G 1C9, Canada; 5 VISN 20 Mental Illness Research, Education and Clinical Center, Seattle, WA 98108, USA; ycho7@uw.edu; 6 Department of Psychiatry and Behavioral Sciences, School of Medicine, University of Washington, Seattle, WA 98195, USA; 7 Division of Pediatric Pulmonology and Sleep Medicine, School of Medicine, University of Washington, Seattle, WA 98195, USA; michelle.sobremonte-king@seattlechildrens.org

**Keywords:** Down syndrome, obstructive sleep apnea, sleep-disordered breathing

## Abstract

Children with Down syndrome (DS) are at high risk of sleep-disordered breathing (SDB). The American Academy of Pediatrics recommends a polysomnogram (PSG) in children with DS prior to the age of 4. This retrospective study examined the frequency of SDB, gas exchange abnormalities, co-morbidities, and surgical management in children with DS aged 2–4 years old at Seattle Children’s Hospital from 2015–2021. A total of 153 children underwent PSG, with 75 meeting the inclusion criteria. The mean age was 3.03 years (SD 0.805), 56% were male, and 54.7% were Caucasian. Comorbidities included (n, %): cardiac (43, 57.3%), dysphagia or aspiration (24, 32.0%), prematurity (17, 22.7%), pulmonary (16, 21.3%), immune dysfunction (2, 2.7%), and hypothyroidism (23, 30.7%). PSG parameter data collected included (mean, SD): obstructive AHI (7.9, 9.4) and central AHI (2.4, 2.4). In total, 94.7% met the criteria for pediatric OSA, 9.5% met the criteria for central apnea, and 9.5% met the criteria for hypoventilation. Only one child met the criteria for hypoxemia. Overall, 60% had surgical intervention, with 88.9% of these being adenotonsillectomy. There was no statistically significant difference in the frequency of OSA at different ages. Children aged 2–4 years with DS have a high frequency of OSA. The most commonly encountered co-morbidities were cardiac and swallowing dysfunction. Among those with OSA, more than half underwent surgical intervention, with improvements in their obstructive apnea hypopnea index, total apnea hypopnea index, oxygen saturation nadir, oxygen desaturation index, total arousal index, and total sleep duration. This highlights the importance of early diagnosis and appropriate treatment. Our study also suggests that adenotonsillar hypertrophy is still a large contributor to upper airway obstruction in this age group.

## 1. Introduction

Down syndrome (DS), or Trisomy 21, is a very common genetic birth defect occurring in about 1 out of 6000 live births [1]. The common features of individuals with DS are delayed psychomotor development, intellectual disability, and hypotonia. Their characteristic physical features include brachycephaly, a flattened nasal bridge, mandibular and maxillary hypoplasia, relative macroglossia, and a narrow nasopharyngeal region [2]. The typical medical comorbidities include congenital defects of the heart; various abnormalities involving the gastrointestinal tract, such as duodenal atresia and tracheoesophageal fistula; airway abnormalities, such as laryngomalacia and tracheomalacia; celiac disease; hearing and vision disorders; hypothyroidism; obesity; and obstructive sleep apnea (OSA) [3]. 

Sleep-disordered breathing (SDB) is highly prevalent among children with DS [4]. OSA is much more prevalent in children with DS compared to healthy children [5]. A large cohort study reported a 66.4% prevalence of OSA in children with DS compared to a 1–4 % prevalence in the population of healthy, typically-developing children [6,7]. A 2018 meta-analysis reported a 76% prevalence of OSA, which included 1200 children with DS [4].

The American Academy of Pediatrics (AAP) recommends referral to a pediatric sleep laboratory for polysomnography (PSG) by the age of 4 years for all children with DS based on the high prevalence of SDB and the known poor correlation between parent-reported symptoms and PSG results [8]. Despite these recommendations, little is known about the clinical practice patterns of screening, identification, and treatment of sleep problems among children with DS. One study of 954 children with DS found that less than half were referred for PSG [9].

There are significant short- and long-term health outcomes associated with unmanaged SDB, such as cardiovascular consequences, including pulmonary hypertension and right heart failure, as well as neurocognitive consequences, including changes in behavior and daytime functioning [10,11,12,13,14,15,16,17].

Craniofacial and airway abnormalities, associated comorbidities, and pulmonary complications place children with DS at a substantially increased risk of having SDB. Patients with DS who have surgical intervention, such as adenotonsillectomy, show an improvement in SDB and sleep parameters [18]. However, subjects with DS frequently had persistent disease and were less likely to benefit from adenotonsillectomy alone [18].

The preschool age group is an important population to study as children undergo rapid physical growth, with adenoid and tonsillar hypertrophy being the primary causes of sleep-disordered breathing [19]. It is also a critical time for brain development. Moreover, current evidence shows improved health and quality of life when treated early [20,21]. 

The purpose of this project was to evaluate the frequency of SDB in children aged 2–4 years with DS who underwent PSG at Seattle Children’s Hospital between 2015 and 2021.

The specific aims are to describe the frequency and severity of SDB in this population, to assess whether the frequency of SDB changes from age 2–4 years, and to describe the frequency of comorbidities in children with DS and their correlation with the severity of SDB in this population. We also sought to determine the frequency of surgical interventions for SDB in this population and whether these interventions improved SDB in this age group.

## 2. Methods

The Seattle Children’s Hospital Institutional Review Board (IRB) approved this project, STUDY#00003376, on 30 September 2021.

This was a retrospective study conducted at a single academic sleep center. The children with DS who come to the center are referred for a sleep study because of sleep complaints and/or have the study done because of the recommended guidelines. Patients aged 2–4 years with DS who completed a diagnostic or split-night PSG at Seattle Children’s Hospital between 2015 and 2021 were included. Exclusion criteria were comorbid genetic or neuromuscular conditions, tracheostomy and ventilator use, surgical intervention for SDB before initial PSG, or less than 2 h of PSG data.

PSGs were performed according to the American Academy of Sleep Medicine (AASM) criteria [22]. If multiple PSGs were completed, the first was chosen. Parameters were measured using Natus SleepWorks PSG software Version 9.2.1 Build 6524 and included electroencephalogram (C3/A2, C4/A1, O1/A2, and O2/A1), electro-oculogram, electromyogram (EMG) of the submentalis muscle, EMG of the right and left tibialis anterior muscles, respiratory signals (nasal pressure transducer, nasal thermistor), effort signals for thorax and abdomen, oximetry, single-lead electrocardiogram (ECG), and video and audio recording. Either capnography (end-tidal CO_2_ [ETCO_2_]), transcutaneous CO_2_ (TcCO_2_), or both were performed. Studies were reviewed and scored by a certified sleep technologist who was blinded to the patient and interpreted by a board-certified sleep physician according to AASM criteria [22]. Respiratory events were classified as obstructive apnea, central apnea, or hypopnea, and the duration and stage (non-rapid eye movement, NREM) sleep stages N1, N2, and N3 or rapid eye movement (REM) of each event were annotated. Metrics of obstructive apnea were: Total apnea hypopnea index (AHI), which is the total number of obstructive apnea; central apnea and hypopnea/total sleep time; obstructive apnea hypopnea index (OAHI), which was the number of obstructive apnea + hypopneas/total sleep time; central apnea index (CAI), which is the number of central apneas/total sleep time; % time spent with CO_2_ levels > 50 mmHg; % time spent with O_2_ saturations < 88%; and O_2_ saturation nadir. OSA was defined as OAHI ≥ 1/h. Severe OSA was defined by OAHI ≥ 10/h. Central sleep apnea (CSA) was defined as CAI ≥ 5/h based on the AASM threshold. Hypoventilation was defined as >25% of time spent with CO_2_ levels > 50 mmHg as measured by ETCO_2_ or TcCO_2_. Hypoxemia was defined as time less than 88% for >5 min.

The total arousal index was calculated as the total number of arousals from spontaneous, respiratory, limb movement, or periodic limb movements divided by the total sleep time.

Airway surgeries performed with the goal of reducing SDB metrics were recorded. These surgeries included adenotonsillectomy, adenoidectomy, tonsillectomy, supraglottoplasty, palatoplasty, and laryngeal cleft repair. If a postoperative PSG was completed, the same parameters as above were collated.

Data were collected from the electronic health record (Epic), and used a secure web-based software for data capture—REDCap version 14.1.2 (Research Electronic Data Capture), hosted at Seattle Children’s Hospital. REDCap is designed for data collection for research studies. It provides validated data through intuitive interface capture, tracking of data changes, and export procedures, and allows data downloads for common statistical analysis and data integration, including external sources.

Summary of data on demographics, PSG results, comorbidities, and surgical revisions of children aged 2–4 years with DS who underwent PSG at Seattle Children’s Hospital between 2015 and 2021 were tabulated. The frequency of OSA at each age, central sleep apnea (CSA), hypoxemia, and hypoventilation were evaluated. The change in the severity of OSA as measured by pre-operative PSG, and post-operative PSG was calculated.

Data were analyzed using the statistical package IBM SPSS 29. Descriptive data included percentages. Comparisons of data before and after intervention were calculated using a *t*-test and significance was considered to be a *p* < 0.05. A correlation matrix was generated to assess the correlation between comorbidities and SDB.

## 3. Results

Demographics: The data presented below are based on 75 children who had PSG (out of 153 children) and met the inclusion criteria. The rest were excluded due to co-morbid genetic or neuromuscular conditions, tracheostomy or ventilator dependence, or surgical intervention for SDB before initial PSG.

The mean age was 3.03 years (SD 0.805), and 56% were male. Overall, 54.7% self-identified as Caucasian, 20% as Hispanic, 13.3% as Asian, 1.3% as Black or African American, 1.3% as Native Hawaiian or Other Pacific Islander, 0% as American Indian, 24% as other or multiple races, and 5.3% declined to answer. These demographics are generally consistent with the demographics of patients who are cared for at Seattle Children’s Hospital (Table 1).

Polysomnogram sleep parameters: The mean sleep efficiency was good, at 85.2% (SD 8.68), and the mean total sleep time was 481.2 min (SD 79.92). The mean percentages of total sleep per sleep stage were N1 sleep 8.4% (SD 6.74), N2 sleep 42.5% (SD 8.1), N3 sleep 29.9% (SD 7.22), and REM sleep 21.5% (SD 14.93), and were unremarkable. The mean arousal index was 13.1 (SD 4.74) (Table 2).

Polysomnogram respiratory parameters and frequency of sleep-disordered breathing: PSG data showed (mean, SD): obstructive AHI (7.9, 9.4) and central AHI (2.4, 2.4). The mean REM AHI was 19.8 (SD 20.36), the mean NREM AHI was lower, at 8.6 (SD 9.56), the mean oxygen saturation during sleep was 96.9% (SD 1.92), the mean nadir O_2_ saturation during sleep was 87.6% (SD 5.48), and the oxygen desaturation index was 7.03 (SD 7.65). The mean percentage of total sleep time for ETCO_2_ was 7.9% (SD 18.2) and for TcCO_2_ was 17.9% (SD 24.46) (Table 3).

**Figure 1:**
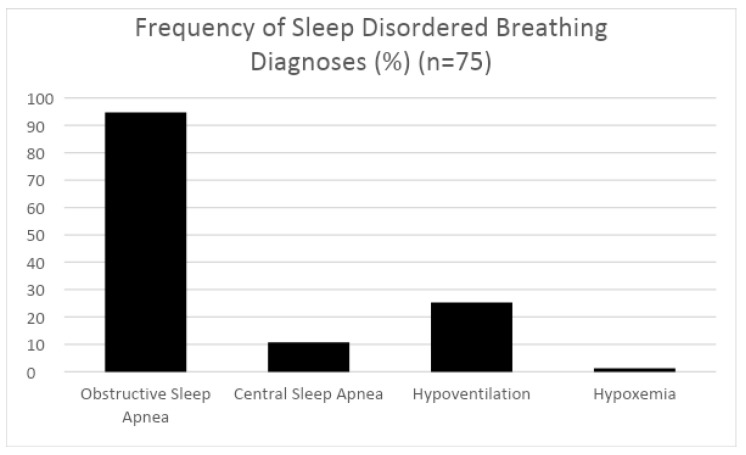
Percentage of study participants pre-operatively diagnosed with sleep-disordered breathing (SDB), including obstructive sleep apnea (OSA), central sleep apnea (CSA), hypoventilation, and hypoxemia.

In total, 94.7% met the criteria for pediatric OSA, 10.7% met the criteria for central apnea, and 25.3% met the criteria for hypoventilation. Only one child (1.3%) met the criteria for hypoxemia (Figure 1).

Age and frequency of obstructive sleep apnea: Using an ANOVA test, there was no statistically significant difference in the frequency of OSA at ages 2, 3, or 4 years (*p* > 0.05). 

**Figure 2:**
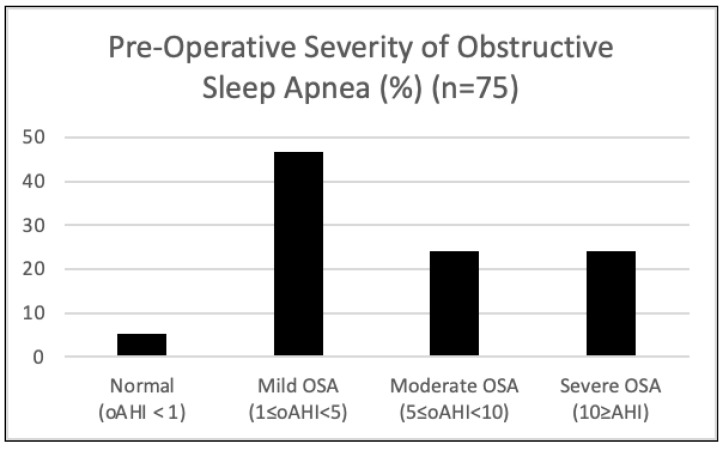
Severity of obstructive sleep apnea (OSA) based on pre-operative polysomnography (PSG) as defined by the American Academy of Sleep Medicine (AASM) pediatric criteria.

Severity of obstructive sleep apnea: Overall, 5.3% of subjects did not have OSA (oAHI < 1), 46.7% had mild OSA (1 ≤ oAHI < 5), 24.0% had moderate OSA (5 ≤ oAHI < 10), and 24.0% had severe OSA (≥10 oAHI). Therefore, in this cohort, 94.7% have OSA (Figure 2).

**Figure 3:**
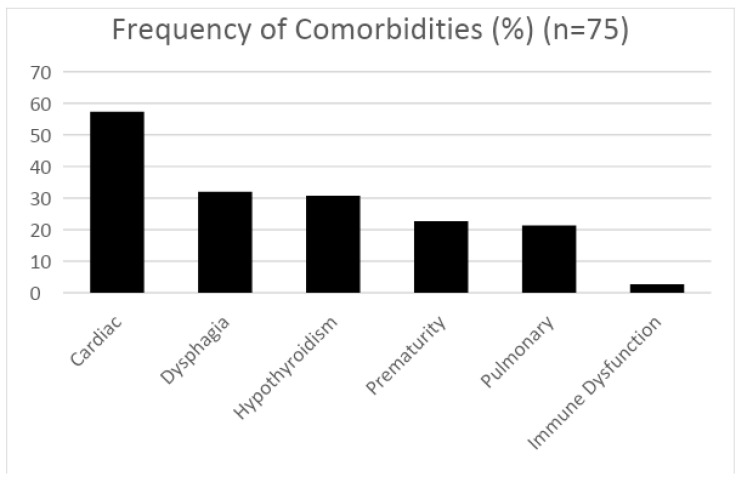
Percentage of study participants diagnosed with common comorbidities of Down syndrome (DS).

Comorbidities of Down syndrome: Comorbidities included cardiac (57.3%), dysphagia or aspiration (32.0%), hypothyroidism (30.7%), prematurity (22.7%), pulmonary (21.3%), and immune dysfunction (2.7%). A correlation matrix of these comorbidities and the severity of OSA and central sleep apnea (CSA) did not show any statistically significant correlation between these variables (*p* > 0.05) (Figure 3).

**Figure 4:**
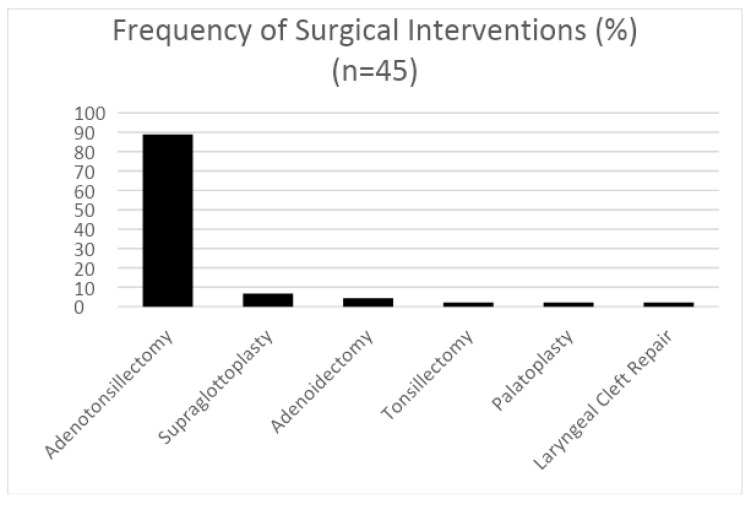
Percentage of study participants who underwent various airway surgeries with the goal of improving their sleep-disordered breathing (SDB). Of the 75 patients enrolled in this study, 45 received surgical intervention.

Surgical intervention for sleep-disordered breathing: A total of 45 of the 75 patients (60%) had surgical intervention for SDB. Of these, 88.9% had adenotonsillectomy, 6.7% had supraglottoplasty, 4.4% had adenoidectomy, 2.2% had tonsillectomy, 2.2% had palatoplasty, and 2.2% had laryngeal cleft repair (Figure 4).

Obstructive sleep apnea before and after surgery: A paired *t*-test of pre-operative OAHI and post-operative OAHI showed that OSA decreased significantly after surgical intervention (*p* = 0.001, 95% CI −11.52, −3.18) (Table 4).

The mean OAHI decreased from 12.1 pre-operatively to 4.8 post-operatively. However, surgery was not curative in all children with DS (Table 4).

While there was no significant difference between pre- and post-operative mean oxygen saturations during sleep, the mean percentage of sleep time O_2_ < 88% and the mean measures of CO_2_ levels (Table 4).

**Figure 5:**
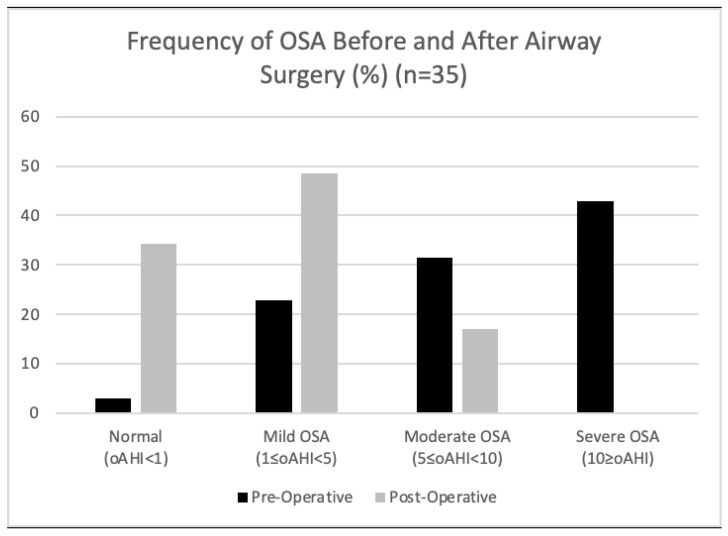
Percentage of study participants with various levels of OSA severity as defined by the American Academy of Sleep Medicine (AASM) pediatric criteria before and after surgical intervention. Of the 45 patients in this study who received a surgical intervention, 35 underwent post-operative polysomnography (PSG).

Only two patients had a higher oAHI after surgery than before. Therefore, 94.3% had an improvement in their OSA (Figure 5). 

Only 2.9% of patients did not have OSA pre-operatively, and 34.3% did not have OSA post-operatively (Figure 5).

Overall, 42.9% of patients had severe OSA pre-operatively and zero patients had severe OSA post-operatively (Figure 5).

Central sleep apnea before and after surgery: A paired t-test of pre-operative and post-operative CAI did not show a significant difference (*p* = 0.63, 95% CI −1.07, 0.66) (Table 4).

Polysomnogram sleep parameters before and after surgery: There was significant difference in the following sleep parameters: A pre-operative mean sleep efficiency of 86.1% (SD 6.69) compared to a post-operative sleep efficiency of 85.5% (SD 8.31) (*p* = 0.013 95% CI −3.49, 2.24), a pre-operative mean total sleep time of 469.5 min (SD 89.36) compared to a longer post-operative mean sleep time of 515.03 min (SD 54.59), a pre-operative mean percentage REM sleep of 20.5% (SD 14.35) compared to a post-operative percentage REM sleep of 19.4% (SD 6.48) (*p* = 0.005 95% CI −6.65, 4.40), and a pre-operative mean arousal index of 14.7 (SD 4.84) compared to a post-operative mean arousal index of 12.5 (SD 4.80) (*p* = 0.0014 95% CI −3.94, −0.47) (Table 5).

There was no significant difference between pre- and post-operative means for the percentage of N1, N2, and N3 sleep (*p* > 0.05) (Table 5).

Other polysomnogram respiratory parameters before and after surgery: There was a significant improvement in the following respiratory parameters: A pre-operative mean REM AHI of 28.8 (SD 26) compared to a post-operative mean REM AHI of 12.2 (SD 8.57) (*p* = 0.0007, 95% CI −25.64, −7.58), a pre-operative mean NREM AHI of 12.0 (SD 11.63) compared to a post-operative mean NREM AHI of 6.5 (SD 5.15) (*p* = 0.013 95% CI −9.70, −1.24), a pre-operative mean oxygen saturation nadir of 85.4% (SD 5.91) compared to a post-operative mean O_2_ saturation nadir of 88.4% (SD 3.97) (*p* = 0.009, 95% CI −8.68, −1.6), and a pre-operative mean ODI of 10.4 (SD 9.59) and a post-operative mean ODI of 5.3 (SD 4.84) (*p* = 0.006 95% CI −8.68, −1.6) (Table 4).

## 4. Discussion

We studied demographic, clinical, and PSG characteristics in preschool children with DS referred for a sleep study. This study showed that the majority (94.7%) of these children with DS had OSA. Overall, 25.3 % of children met the criteria for hypoventilation and 10.7% had central apnea. Only 5.3% of patients in this study group had no evidence of OSA during the sleep study. This implies that there is a high frequency of SDB, especially OSA, in children aged 2–4 years with Down syndrome. The frequency of SDB does not seem to vary by age and remains statistically similar at ages 2, 3, and 4 years. This is in keeping with the growth of the tonsils and adenoids in these young children.

Nocturnal hypoventilation was also observed in 25.3 % of our study population. The presence of nocturnal hypoventilation (ETCO_2_ > 50 mm Hg during 25% of total sleep time, as defined by AASM) in our study is consistent with previously reported studies in children with DS [23,24]. One study showed nocturnal hypoventilation was present in 18% of DS, and another study by Fan et al. showed 22% had nocturnal hypoventilation in a cohort that included 144 PSG recordings of adults and children [23,25]. Our study showed that nocturnal hypoventilation can occur at a young age in children with DS.

The predisposition to develop OSA and nocturnal hypoventilation in children with DS in this age group is multifactorial. Children with DS tend to have craniofacial abnormalities, such as mandibular or maxillary hypoplasia, which may cause a reduction in oral cavity volume [3,26,27]. Children with DS also have upper airway abnormalities such as narrow nasopharynx, shortened palate, laryngomalacia, and tracheomalacia [28,29]. They tend to have adenotonsillar hypertrophy and relative macroglossia in a smaller oral cavity. Tonsillar enlargement generally starts in this age group. Generalized hypotonia, or overall low neuromuscular tone, is also seen in children with DS. The poor upper airway tone can make children with DS prone to glossoptosis, laryngomalacia, and hypopharyngeal collapse. These factors are also worsened by a predisposition towards obesity—another significant risk factor for the development of OSA [30]. Regardless of the pathophysiology, this study emphasizes the importance of screening for OSA in children with DS, starting in early childhood. 

The prevalence of atopic disease (eczema, asthma, rhinitis, eosinophilic esophagitis, etc.) in this cohort is not known. The management of mild OSA can include intranasal steroids and montelukast, either to help reduce the need for surgery or for use post-operatively in otherwise healthy children. We are not able to comment on whether there is a role for the use of these medications in children with DS. One study evaluated the role of intranasal steroids and/or montelukast in a small group of children (N = 45) with DS with a median age of 7.4 years, and no effect of the medication was found, suggesting that the etiology of OSA in DS may be more structural than inflammatory [31]. The comorbidities in our study were cardiac (57.3%), dysphagia (32%), hypothyroidism (30.7%), prematurity (22.7%), and pulmonary (21.3%). Most of these comorbidities could also contribute to the higher prevalence of OSA.

Previous work published by our group showed that in infants with DS, dysphagia was correlated with the severity of OSA [32]. However, comorbidities did not appear to influence the presence or severity of OSA in the 2–4 year age group in this study. This may be due to the small sample size not achieving statistical significance (power analysis was not performed). It may be secondary to the increased contribution of adenotonsillar hypertrophy in this age group or because the disease process may evolve throughout the development of children with DS. The hypotonia could play a role in this age group. There could be multiple factors impacting each age group, such as increasing rates of obesity in older children. 

Therefore, as pediatric patients with Down syndrome are more at risk of having OSA, timely diagnosis and treatment is important. The first line of standard treatment are adenoidectomy and tonsillectomy for pediatric patients with OSA who have tonsillar and adenoid hypertrophy. Upper airway surgery, specifically adenotonsillectomy, has an approximately 80% success rate in treating otherwise healthy children with OSA with adenoid and tonsillar hypertrophy, according to the AAP and the American Academy of Otolaryngology Head and Neck Surgery [33]. There are, however, only a few studies available in the DS population, with these often having small sample sizes, especially in the 2–4 year old age group. 

Over half of the children in this study received surgical intervention for OSA. Almost all patients had an improvement in their OSA post-surgery, especially in children with severe OSA, implying that tonsillar and adenoid hypertrophy are important in the pathophysiology of OSA in this group. This supports previous findings that showed tonsillar hypertrophy in DS is a predictor of OSA in children younger than 3 years of age [34].

Given the complexity of risk factors in DS, it is likely that other factors than tonsillar and adenoid hypertrophy were contributing to OSA in some children in this study group. The need for other types of airway surgical intervention that were performed in some children in this study suggests that it is caused by other factors. 

Multiple studies have also shown similar findings. Maris et al. studied 34 young children with DS (mean age of 4 years old), the majority whom had severe OSA and showed significant improvement in their oAHI and an increase in their minimum oxygen saturation post-adenoidectomy and tonsillectomy. However, almost half of the children had persistent OSA, which was not correlated to age, gender, or BMI z-score [35]. In Sweden, Nerfeldt et al. studied a slightly older patient population (mean age of 8 years old) with DS and OSA. A total of 33 patients in their non-systematic subgroup underwent post-operative PSG (adenoidectomy, tonsillectomy, and adenopharyngoplasty), with a residual prevalence of moderate to severe OSA of 63.6%, but values were significantly improved as a whole as the median AHI dropped from 21.1 to 12.4 and the median OSA-18 rating improved from 54 to 35 [36].

Ingram et al. studied 75 children with DS who had an adeno-tonsillectomy or a tonsillectomy with a previous adenoidectomy. They observed that the AHI at baseline was 21.3 ± 19.7, and, after six months, it had decreased to 8.0 ± 8.1 [37]. Another study conducted by Sudarasaan et al. in 37 children with either DS or mucopolysaccharidosis (MPS) also found a decrease in the AHI from a baseline of 3.83 ± 1.36 to 2.62 ± 0.87 at six months post-operatively, suggesting that surgery did reduce the number of events but not necessarily cure the children of OSA. However, it is difficult to ascertain the true clinical benefit of AT in this study since the patients had a low baseline AHI [38]. Another study by Shete et al. compared the AHI and REM-AHI before and after AT in children with DS vs. those without DS. They found that for both cohorts of children, AT did not significantly affect the sleep stage distribution and sleep stage percentages, the sleep efficiency (ratio of total sleep time to time in bed), or the mean arousal index (number of arousals per hour of total sleep time). For the cohort of children with DS who underwent AT, 55% required positive airway pressure ventilation, and 18% required nocturnal oxygen. In contrast, none of the children without DS required any additional treatment after AT [39].

Similarly to the results we found in our study, these studies also show that despite improvements in their oAHI, surgical therapy is not curative for all children with DS, and the cure rate is much lower than the reported cure rate in otherwise healthy children.

This suggests that in children with DS, OSA is in part due to other anatomical structures beyond the adenoid and tonsil, and other functions of muscles are involved in the airway. As such, it should be noted that other anatomical factors beyond adenoid/tonsil structures should be considered for targeted surgical planning.

Hypotonia is also a major factor in DS but is not clinically measurable. This could also be the potential explanation for residual disease. The role of DISE in these children would be helpful, especially for any residual disease after adenotonsillectomy. 

These findings further support the need for routine screening with PSG pre- and post-surgical therapy to perform other appropriate medical and surgical interventions post-adenoidectomy and tonsillectomy. 

The limitations of our study include reporting from one academic center, PSG findings only in a small cohort of DS children, and one specific time frame without longitudinal data. Clinical data were not reviewed to determine if there was any correlation between symptoms and PSG. By nature of the retrospective design of the study, not all children were studied in a systematic manner. Thus, a selection bias exists in that the prevalence of OSA found in this study is likely overrated since all children included had been referred with a high pre-test likelihood of SDB. We did not study all children with DS, so the increased prevalence may be skewed because in this age group, there are more likely to be recurrent respiratory infections and proportionately larger adenoids and tonsils compared to older children with DS. Despite this limitation, this study highlights the excessive burden of SDB in toddler and preschool children with DS and the diversity of presenting comorbidities. Although OSA significantly improved after surgical intervention over a short-term period, long-term post-surgical outcomes (OSA) and DS-related outcomes such as neurocognition are unknown. Future studies should address these gaps. 

In conclusion, preschool children with DS frequently have SDB, especially OSA. Many children with DS and OSA had surgical intervention and a subsequent cure of or improvement in their OSA, further highlighting the importance of early diagnosis and appropriately targeted treatment. Based on the results of this study, young children of preschool age with DS should be referred for PSG, regardless of comorbidities or reported symptoms.

## Figures and Tables

**Table 1:** Demographic data (*n* = 75).

**Race**	**%**
American Indian	0.0
Asian	13.3
Black or African American	1.3
Native Hawaiian or Other Pacific Islander	1.3
White	54.7
Other or multiple races	24.0
Declined to answer	5.3
**Ethnicity**	**%**
Hispanic	20.0
Non-Hispanic	74.7
Declined to answer	5.3

**Table 2:** Polysomnogram sleep parameters.

	Mean (SD)	*n*
Sleep efficiency (%)	85.2% (8.68)	75
Total sleep time (min)	481.2 (79.92)	75
Stage N1 % of sleep time	8.4% (6.74)	75
Stage N2 % of sleep time	42.5% (8.1)	74
Stage N3 % of sleep time	29.9% (7.22)	75
Stage REM % of sleep time	21.5% (14.93)	75
Total arousal index	13.1 (4.74)	75

**Table 3:** Polysomnogram respiratory parameters.

Respiratory Parameters	Mean (SD)	*n*
Total AHI	10.30 (10.20)	75
OAHI	7.9 (9.40)	75
CAI	2.40 (2.40)	75
REM AHI	19.8 (20.36)	74
NREM AHI	8.6 (9.56)	73
Mean O_2_ saturation during sleep	96.9% (1.92)	75
Nadir O_2_ saturation during sleep	87.6% (5.48)	75
% Total sleep time O_2_ < 88%	0.8% (6.16)	75
Oxygen desaturation index	7.03 (7.65)	75
% Total sleep time ETCO_2_ > 50 mmHg	7.9% (18.2)	48
% Total sleep time TcCO_2_ > 50 mmHg	17.9% (24.46)	45

**Table 4:** Pre- and post-surgery polysomnogram respiratory parameters.

Respiratory Parameters	Pre-Surgery Mean (SD)	Post-Surgery Mean (SD)	* *p* Value (95% CI)	*n*
Total AHI	14.9 (12.63)	7.4 (5.42)	0.0014 (−11.98, −3.143)	35
OAHI	12.1 (11.99)	4.8 (3.72)	0.001 (−11.52, −3.18)	35
CAI	2.8 (2.69)	2.6 (2.49)	0.63 (−1.07, 0.66)	35
REM AHI	28.8 (26)	12.2 (8.57)	0.0007 (−25.64, −7.58)	34
NREM AHI	12.0 (11.63)	6.5 (5.15)	0.013 (−9.70, −1.24)	34
Mean O_2_ saturation	96.2% (2.43)	96.9% (1.37)	0.15 (−0.23, 0.9)	35
O_2_ saturation nadir	85.4% (5.91)	88.4% (3.97)	0.009 (0.78, 5.14)	35
% Sleep time O_2_ < 88%	1.6% (9.01)	0.02% (0.05)	0.3 (−4.68, 1.5)	35
ODI	10.4 (9.59)	5.3 (4.84)	0.006 (−8.68, −1.6)	35
% Sleep time ETCO_2_ > 50 mmHg	12.9% (27.33)	7.2% (17.41)	0.34 (−18.38, 6.88)	14
% Sleep time TcCO_2_ > 50 mmHg	16.7% (22.4)	12.2% (23.97)	0.58 (−21.36, 12.27)	19

* Paired *t*-test.

**Table 5:** Pre- and post-surgery polysomnogram sleep parameters.

Sleep Parameters	Pre-Surgery Mean (SD)	Post Surgery Mean (SD)	* *p* Value (95% CI)	*n*
Sleep efficiency (%)	86.1% (6.69)	85.5% (8.31)	0.013 (−3.49, 2.24)	35
Total sleep rime (min)	469.5 (89.36)	515.03 (54.59)	0.013 (10.39, 80.6)	35
Stage N1 % of sleep time	9.8% (7.19)	7.7% (6.02)	0.17 (−5.1, 0.96)	35
Stage N2 % of sleep time	42.0% (9.3)	43.3% (8.59)	0.49 (−2.49, 5.11)	34
Stage N3 % of sleep time	30.5% (8.74)	29.1% (9.99)	0.53 (−5.48, 2.85)	35
Stage REM % of sleep time	20.5% (14.35)	19.4% (6.48)	0.005 (−6.65, 4.40)	35
Total arousal index	14.7 (4.84)	12.5 (4.80)	0.014 (−3.94, −0.47)	35

* Paired *t*-test.

## Data Availability

The data presented in this study are available on request from the corresponding author. The data are not publicly available due to patient privacy.
